# Wide field imaging of van der Waals ferromagnet Fe_3_GeTe_2_ by spin defects in hexagonal boron nitride

**DOI:** 10.1038/s41467-022-33016-2

**Published:** 2022-09-13

**Authors:** Mengqi Huang, Jingcheng Zhou, Di Chen, Hanyi Lu, Nathan J. McLaughlin, Senlei Li, Mohammed Alghamdi, Dziga Djugba, Jing Shi, Hailong Wang, Chunhui Rita Du

**Affiliations:** 1 Department of Physics, University of California, San Diego, La Jolla, CA 92093 USA; 2 Department of Physics, University of Houston, Houston, TX 77204 USA; 3 Texas Center for Superconductivity, University of Houston, Houston, TX 77204 USA; 4 Department of Physics and Astronomy, University of California, Riverside, CA 92521 USA; 5 Center for Memory and Recording Research, University of California, San Diego, La Jolla, CA 92093 USA

**Keywords:** Imaging techniques, Two-dimensional materials, Magnetic properties and materials, Optical materials and structures

## Abstract

Emergent color centers with accessible spins hosted by van der Waals materials have attracted substantial interest in recent years due to their significant potential for implementing transformative quantum sensing technologies. Hexagonal boron nitride (hBN) is naturally relevant in this context due to its remarkable ease of integration into devices consisting of low-dimensional materials. Taking advantage of boron vacancy spin defects in hBN, we report nanoscale quantum imaging of low-dimensional ferromagnetism sustained in Fe_3_GeTe_2_/hBN van der Waals heterostructures. Exploiting spin relaxometry methods, we have further observed spatially varying magnetic fluctuations in the exfoliated Fe_3_GeTe_2_ flake, whose magnitude reaches a peak value around the Curie temperature. Our results demonstrate the capability of spin defects in hBN of investigating local magnetic properties of layered materials in an accessible and precise way, which can be extended readily to a broad range of miniaturized van der Waals heterostructure systems.

## Abstract (web summary)

Hexagonal boron nitride (h-BN) has been used extensively to encapsulate other van der Waals materials, protecting them from environmental degradation, and allowing integration into more complex heterostructures. Here, the authors make use of boron vacancy spin defects in h-BN using them to image the magnetic properties of a Fe_3_GeTe_2_ flake.

## Introduction

Optically active spin defects in wide band-gap semiconductors promise to enable a broad range of emerging applications in quantum information sciences and technologies^1–4^. To date, nitrogen-vacancy centers in diamond^1,2^, as well as divacancy and silicon-vacancy centers in silicon carbide^3–5^, have been among the most prominent candidates, and have been successfully applied to quantum sensing, computing, and network research, enabling unprecedented field sensitivity, spatial resolution, and state-of-the-art spin-qubit operations^2,6,7^. Many of these advantages derive from the quantum-mechanical nature of these spin defects, which are endowed with excellent quantum coherence, single-spin addressability, and remarkable functionality over a broad temperature range^1,3–5,8,9^.

More recently, the flourishing catalog of van der Waals materials^10^ has provided a diverse new playground to enrich this field. There is ongoing and intense activity to explore emergent spin defects and color centers in atomic layers of van der Waals crystals, e. g. transition metal dichalcogenides MoS_2_^11,12^, WSe_2_^12,13^, and hexagonal boron nitride (hBN)^12,14–28^. In comparison with their conventional counterparts imbedded in three-dimensional solid-state-media, spin defects hosted by two-dimensional (2D) materials exhibit improved versatility for implementing ultrasensitive quantum sensing of proximate objects and remarkable compatibility to device integration^14,26^. For instance, hexagonal boron nitride (hBN), one of the most intensively studied candidates, has been widely employed as an encapsulation layer and gate dielectric material in fabricating functional 2D devices^29–32^. Thus, nanoscale proximity between spin defects in a hBN thin sheet and a layered 2D material can be readily established in van der Waals heterostructures, offering a previously unexploited quantum sensing platform to explore the local physical quantities of interest in an accessible and precise way.

Despite these potential benefits and many pioneering studies, to date, experimental demonstration of quantum microscopy using spin defects in a real van der Waals heterostructure remains a formidable challenge. In this work, we report nanoscale quantum sensing and imaging of exfoliated 2D ferromagnet Fe_3_GeTe_2_ (FGT) flakes^33–41^ by boron vacancy ${V}_{{{{{{\rm{B}}}}}}}^{-}$ spin defects in an adjacent hBN capping layer. Exploiting a wide-field magnetometry method^42–46^, we directly image the local magnetic texture of the FGT flake and its characteristic temperature and field dependent magnetization evolution behavior. Taking advantage of spin relaxometry techniques^47–51^, we have observed spatially varying spin fluctuations in the FGT flake, whose magnitude reaches a peak value around the Curie temperature, consistent with the expected ferromagnetic phase transition. We highlight that the presented quantum sensing platform built on spin defects in van der Waals crystals can be extended naturally to a large family of miniaturized 2D heterostructure systems^10,52,53^, bringing new opportunities for investigating the local spin, charge, and thermal properties of emergent quantum materials and devices.

## Results

**Fig. 1:**
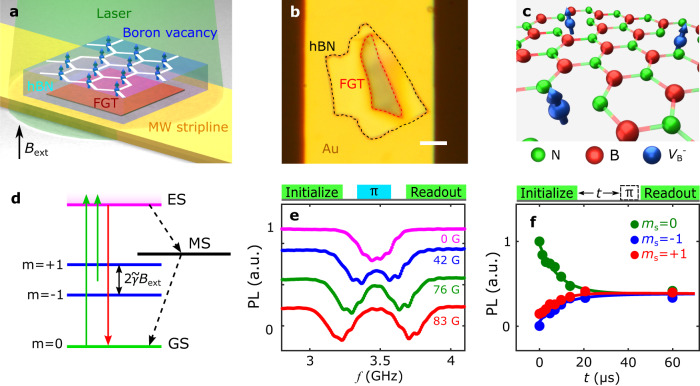
Quantum sensing using ${V}_{{{{{{\rm{B}}}}}}}^{-}$ spin defects in hexagonal boron nitride (hBN). **a** Schematic of a Fe_3_GeTe_2_(FGT)/hBN van der Waals heterostructure transferred onto an Au microwave stripline for wide-field magnetometry measurements. **b** Optical microscope image of a prepared FGT/hBN device. The FGT and hBN flakes are outlined with red and black dashed lines, respectively. The scale bar is 5 ${{{{{\rm{\mu }}}}}}$m. **c** Schematic of ${V}_{{{{{{\rm{B}}}}}}}^{-}$ spin defects (blue arrows) formed in a hexagonal crystalline structure with alternating boron (red) and nitrogen (green) atoms. A negatively charged boron atom vacancy ${V}_{{{{{{\rm{B}}}}}}}^{-}$ is surrounded by three nitrogen atoms located in the nearest neighboring sites. **d** Energy level diagram of a ${V}_{{{{{{\rm{B}}}}}}}^{-}$ spin defect and schematic illustration of optical excitation (green arrow), radiative recombination (red arrow), and nonradiative decay (black dotted arrow) processes between the ground state (GS), excited state (ES), and metastable state (MS). **e** Top panel: optical and microwave sequence of pulsed optically detected magnetic resonance (ODMR) measurements. Bottom panel: ODMR spectra of ${V}_{{{{{{\rm{B}}}}}}}^{-}$ spin defects measured at a series of perpendicularly applied external magnetic fields *B*_ext_. **f** Top panel: optical and microwave sequence of spin relaxometry measurements. Bottom panel: a set of spin relaxometry data of ${V}_{{{{{{\rm{B}}}}}}}^{-}$ spin defects showing spin dependent photoluminescence measured as a function of delay time *t*. The external magnetic field is 185 G applied along the out-of-plane direction, and the measurement temperature is 295 K.

Before discussing the details of our experimental results, we first review our measurement platform and device structure, as illustrated in Fig. 1a. We exfoliated an FGT flake and mechanically transferred it onto a patterned Au microwave transmission line. Following this, we encapsulated the sample with an hBN layer. The device preparation process was performed in a glove box with argon environment to minimize environmental effects (see Methods for details). An optical microscope image shown in Fig. 1b provides an overview of a prepared device, where the thickness of the FGT and hBN flakes were characterized by atomic force microscopy (see Supplementary Information Note 1 for details). Boron vacancy ${V}_{{{{{{\rm{B}}}}}}}^{-}$ spin defects in the hBN flake were created by Helium ion implantation with an energy of 5 keV and a dose of 5 $\times$ 10^13^ cm^−2^. Figure 1c illustrates the structure of ${V}_{{{{{{\rm{B}}}}}}}^{-}$ in a hexagonal crystalline structure with alternating boron (red) and nitrogen (green) atoms, where three nitrogen atoms are adjacent to each boron atom vacancy ${V}_{{{{{{\rm{B}}}}}}}^{-}$. The negatively charged ${V}_{{{{{{\rm{B}}}}}}}^{-}$ spin defect has an *S* = 1 electron spin and serves as a three-level quantum system, as shown in Fig. 1d. In the present study, we used microwave currents flowing in the Au microwave transmission line to control the quantum spin state of ${V}_{{{{{{\rm{B}}}}}}}^{-}$ spin defects, which can be optically accessed via spin-dependent photoluminescence (PL). The Au underlayer also enhances the PL and optical contrast of ${V}_{{{{{{\rm{B}}}}}}}^{-}$ spin defects^16^, aiding the quantum microscopy measurements discussed below. The distance between the ${V}_{{{{{{\rm{B}}}}}}}^{-}$ defect centers and the top surface of the FGT sample is estimated to be ~50 nm based on Stopping and Range of Ions in Matter (SRIM) simulations. Exfoliated FGT flakes show spontaneous perpendicular magnetization due to reduced crystal symmetry of the layered structure^33,35,36^. We have prepared separate devices to systematically and reproducibly characterize the magneto-transport properties of exfoliated FGT flakes, whose Curie temperature is measured to be ~200 K, in qualitative agreement with previous studies (see Supplementary Information Note 2 for details)^33,35,40^.

We now utilize wide-field microscopy to demonstrate optically detected magnetic resonance (ODMR) and spin relaxation of ${V}_{{{{{{\rm{B}}}}}}}^{-}$ defect centers (see Methods for details). The top panel of Fig. 1e shows the optical and microwave measurement sequence. We utilize 1-${{{{{\rm{\mu }}}}}}$s-long green laser pulses for spin initialization and readout, and ~100-ns-long microwave $\pi$ pulses^54^ to induce spin transitions of ${V}_{{{{{{\rm{B}}}}}}}^{-}$ spin defects. We sweep the frequency *f* of the microwave $\pi$ pulses and measure the fluorescence across the field of view projected on a CMOS camera. When *f* matches the electron spin resonance (ESR) frequencies, ${V}_{{{{{{\rm{B}}}}}}}^{-}$ spin defects are excited to the m_s_ = $\pm$1 states, which are more likely to relax through a non-radiative pathway back to the m_s_ = 0 ground state and emit reduced PL. The bottom panel of Fig. 1e shows a series of ODMR spectra of ${V}_{{{{{{\rm{B}}}}}}}^{-}$ defect centers measured at room temperature with different external magnetic fields *B*_ext_ applied along the out-of-plane direction. For *B*_ext_ = 0, the energy level of the m_s_ = $\pm$1 states of ${V}_{{{{{{\rm{B}}}}}}}^{-}$ spin defects exhibit a small separation of ~100 MHz due to the off-axial zero field splitting effect^14,26,55^ and the average ESR frequency equals ~3.47 GHz at room temperature^14,26,28,55^. For *B*_ext_ $>$ 0, the Zeeman coupling separates the m_s_ = $-$1 and m_s_ = $+$1 spin states by an energy gap of magnitude 2$\widetilde{\gamma }$*B*_ext_ (see Supplementary Information Note 3 for details), where $\widetilde{\gamma }$ denotes the gyromagnetic ratio of ${V}_{{{{{{\rm{B}}}}}}}^{-}$ defect centers. To characterize the quantum coherence of ${V}_{{{{{{\rm{B}}}}}}}^{-}$ spin defects, we perform spin relaxometry measurements with a measurement protocol shown in the top panel of Fig. 1f. A microsecond scale green laser pulse is first applied to initialize the ${V}_{{{{{{\rm{B}}}}}}}^{-}$ spin defects to the m_s_ = 0 state. During the time delay, fluctuating magnetic fields at the ESR frequencies will accelerate spin relaxation of ${V}_{{{{{{\rm{B}}}}}}}^{-}$ spin defects. After a delay time *t*, we measure the occupation probabilities of the ${V}_{{{{{{\rm{B}}}}}}}^{-}$ spin defects at the m_s_ = 0 and $\pm$1 states by applying a microwave $\pi$ pulse on the corresponding ESR frequencies and measuring the spin-dependent PL by a green-laser readout pulse. The bottom panel of Fig. 1f shows the integrated PL intensity of the m_s_ = 0 and $\pm$1 states measured as a function of the delay time *t*. By fitting the data with a three-level model^51,56^, the spin relaxation rate ${\Gamma }_{0}$ of ${V}_{{{{{{\rm{B}}}}}}}^{-}$ spin defects is obtained to be 39 KHz (38 KHz) for m_s_ = 0 $\to -$1 (+1) transition at 295 K, consistent with previous studies^16,21,55^.

**Fig. 2:**
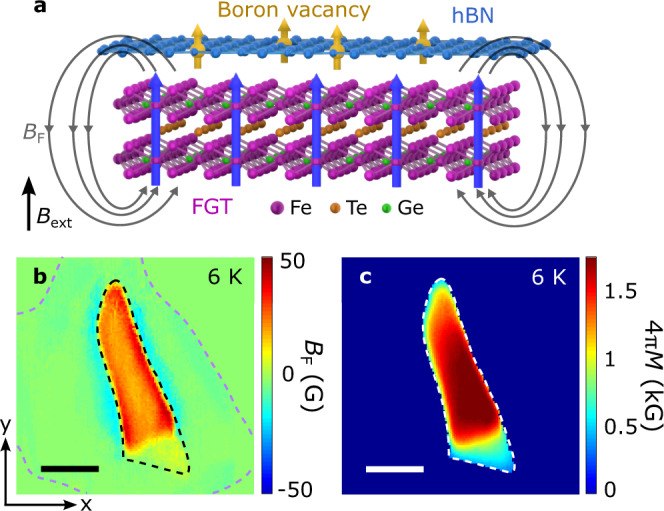
Wide-field imaging of magnetization of an exfoliated FGT flake by ${V}_{{{{{{\rm{B}}}}}}}^{-}$ spin defects in hBN. **a** Schematic illustration of quantum sensing of local stray fields *B*_F_ generated from FGT by proximate ${V}_{{{{{{\rm{B}}}}}}}^{-}$ spin defects. **b, c** Two-dimensional maps of static stray field *B*_F_ (**b**) and reconstructed magnetization 4$\pi$*M* (**c**) of an exfoliated FGT flake measured at 6 K with an external perpendicular magnetic field *B*_ext_ of 142 G. The black and purple dashed lines in Fig. 2b outline the boundary of the FGT and hBN flake, respectively, and the scale bar is 5 ${{{{{\rm{\mu }}}}}}$m.

After demonstrating the ODMR and spin relaxometry measurement capabilities, next, we use ${V}_{{{{{{\rm{B}}}}}}}^{-}$ spin defects in hBN to directly image magnetic textures of an exfoliated FGT flake. Wide-field magnetometry exploits the Zeeman splitting effect of the ensembles of ${V}_{{{{{{\rm{B}}}}}}}^{-}$ defect centers to measure the local magnetic stray fields generated from the proximate FGT flake, as illustrated in Fig. 2a. It is worth noting that the ${V}_{{{{{{\rm{B}}}}}}}^{-}$ spins are naturally orientated along the out-of-plane direction^14^, serving as an ideal local sensor to investigate the magnetic dynamics and phase transition of FGT with spontaneous perpendicular anisotropy. The magnitude of the local static magnetic field *B*_tot_ can be extracted as follows: *B*_tot_ = $\pi \triangle {f}_{{{{{{\rm{ESR}}}}}}}/\widetilde{\gamma }$, where $\triangle {f}_{{{{{{\rm{ESR}}}}}}}$ characterizes the Zeeman splitting of the ${V}_{{{{{{\rm{B}}}}}}}^{-}$ spin defects. Subtracting the contribution from the external magnetic field *B*_ext_, the magnetic stray field *B*_F_ generated from the FGT flake can be quantitatively measured. By performing spatially dependent ODMR measurements over the ${V}_{{{{{{\rm{B}}}}}}}^{-}$ spin ensembles, we are able to obtain a 2D stray field map as shown in Fig. 2b, which is measured at a temperature *T* = 6 K and an external magnetic field *B*_ext_ = 142 G. Through well-established reverse-propagation protocols^46,57,58^ (see Supplementary Information Note 3 for details), we can reconstruct the corresponding magnetization 4*πM* map of the FGT flake, as shown in Fig. 2c. The spatially averaged magnetization of the FGT flake is 1.3 kG at 6 K, in qualitative agreement with the bulk value^33^. The variation of the local magnetization could result from inhomogeneities, magnetic domains, or localized defects^39,59^.

**Fig. 3:**
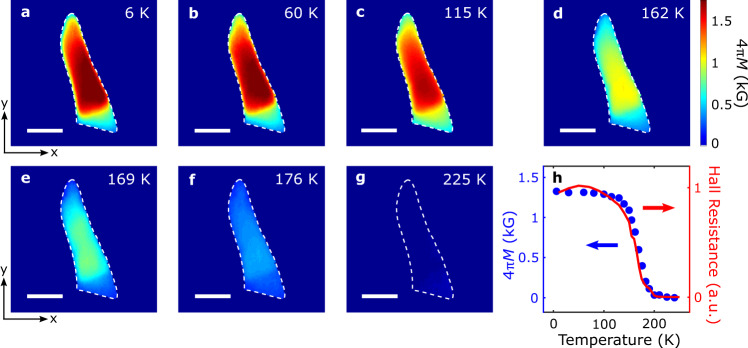
Quantum imaging of temperature dependence of FGT magnetization. Reconstructed magnetization (4*πM*) maps of the FGT flake at *B*_ext_ = 142 G and temperatures of 6 K (**a**), 60 K (**b**), 115 K (**c**), 162 K (**d**), 169 K (**e**), 176 K (**f**), and 225 K (**g**), respectively. The white dashed lines outline the boundary of the exfoliated FGT flake, and the scale bar is 5 ${{{{{\rm{\mu }}}}}}$m. **h** Temperature dependence of spatially averaged magnetization of the FGT flake (blue points), in agreement with the variation behavior of the normalized Hall resistance presented in arbitrary units (a. u.) (red curve). The magneto-transport results were measured in a separate FGT flake with similar thickness.

**Fig. 4:**
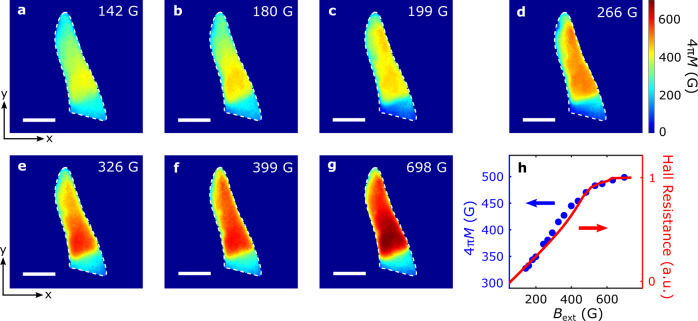
Quantum imaging of field dependence of FGT magnetization. Reconstructed magnetization (4*πM*) maps of the FGT flake measured at 178 K with an external perpendicular magnetic field *B*_ext_ of 142 G (**a**), 180 (**b**), 199 G (**c**), 266 G (**d**), 326 G (**e**), 399 G (**f**), and 698 G (**g**), respectively. The white dashed lines outline the boundary of the FGT flake, and the scale bar is 5 ${{{{{\rm{\mu }}}}}}$m. **h** Field dependence of spatially averaged magnetization of the FGT flake (blue points), consistent with the variation behavior of the normalized Hall resistance presented in arbitrary units (a. u.) (red curve). The magneto-transport results were measured in a separate FGT flake with similar thickness.

We now present systematic wide-field magnetometry results to directly image the magnetic phase transition of the FGT flake across the Curie temperature. Figure 3a–g show the reconstructed magnetization maps of the FGT flake measured with temperatures varying from 6 to 225 K and an external perpendicular magnetic field *B*_ext_ of 142 G. In the low temperature regime (*T* $<$ 100 K), the exfoliated FGT flake exhibits robust magnetization, as shown in Fig. 3a, b, indicating a long-range ferromagnetic order sustained by the intrinsic magnetocrystalline anisotropy of FGT. The measured FGT magnetization decreases with increasing temperature due to enhanced thermal fluctuations (Fig. 3c). When approaching the Curie temperature, the energy of thermal fluctuations becomes comparable to the exchange energy of FGT, resulting in significant suppression of the FGT magnetization (Fig. 3d). Further increasing of the temperature leads to shrinking of the magnetic domain and decreasing magnetization of the FGT flake (Fig. 3e, f). Above the Curie temperature (*T* = 225 K), measured magnetization disappears over the entire FGT flake area (Fig. 3g). Figure 3h summarizes the temperature-dependent evolution of the spatially averaged magnetization of the FGT flake. The magnetic moment of the FGT flake exhibits a gradual decay in the low temperature regime, followed by a dramatic drop during the ferromagnetic phase transition, in agreement with the magneto-transport characterization results (see Supplementary Information Note 2 for details). To further highlight the evolution of magnetic domains in the FGT flake, we present wide-field magnetometry results under different external magnetic fields. Figure 4a–g show a series of magnetization maps of the FGT flake measured with *B*_ext_ varied from 142 G to 698 G at a fixed temperature of 178 K. Qualitatively, the FGT magnetization increases with increasing *B*_ext_ and reaches a saturation value when *B*_ext_ $\ge$ 600 G. This is accompanied by the propagation of magnetic domain walls and expansion of magnetic domain taking place at the nanoscale as shown in the presented images. Figure 4h shows field dependence of the spatially averaged FGT magnetization, consistent with the variation of the anomalous Hall resistance measured in the same magnetic field regime (see Supplementary Information Note 2 for details). We note that the previously observed labyrinthine magnetic domains in FGT are not visible here due to the optical diffraction dictated spatial resolution limit^35^.

**Fig. 5:**
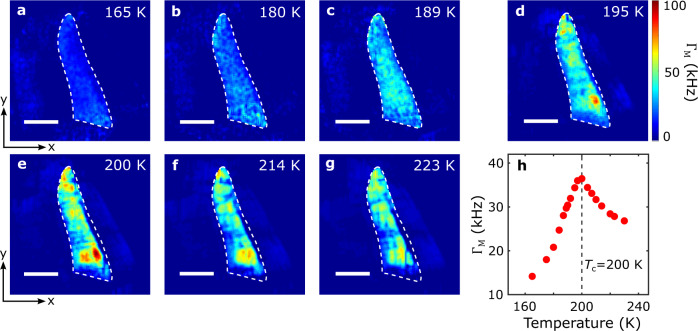
Quantum imaging of spin fluctuations in an exfoliated FGT flake. **a** Spin relaxation maps of ${V}_{{{{{{\rm{B}}}}}}}^{-}$ spin defects measured at temperatures of 165 K (**a**), 180 K (**b**), 189 K (**c**), 195 K (**d**), 200 K (**e**), 214 K (**f**), and 223 K (**g**), respectively. The ESR frequency of ${V}_{{{{{{\rm{B}}}}}}}^{-}$ spin defects ${f}_{{{{{{\rm{ESR}}}}}}}$ is set to be approximately 1.9 GHz in these measurements with an external magnetic field *B*_ext_ = 590 G. The white dashed lines outline the boundary of the FGT flake, and the scale bar is 5 ${{{{{\rm{\mu }}}}}}$m. **h** Temperature dependence of the spatially averaged spin relaxation rate ${\Gamma }_{{{{{{\rm{M}}}}}}}$ of ${V}_{{{{{{\rm{B}}}}}}}^{-}$ spin defects located directly above the FGT flake. The black dashed line marks the Curie temperature of the FGT flake.

In addition to sensing static magnetic stray fields, the excellent quantum coherence of ${V}_{{{{{{\rm{B}}}}}}}^{-}$ spin defects in hBN also provides the opportunity for probing fluctuating magnetic fields that are challenging to access by conventional magnetometry methods^47,48,50,51,55^. Lastly, we utilize the spin relaxometry method demonstrated above to probe the temperature dependence of the magnetic fluctuations in the FGT flake, revealing the intriguing physics underlying the longitudinal magnetic susceptibility and diffusive spin transport properties^60,61^. Fig. 5a–g show a series of ${V}_{{{{{{\rm{B}}}}}}}^{-}$ spin relaxation rate maps measured with temperatures between 165 K and 223 K. Note that the background of the intrinsic relaxation rate of ${V}_{{{{{{\rm{B}}}}}}}^{-}$ has been subtracted to highlight the contribution ${\Gamma }_{{{{{{\rm{M}}}}}}}$ from the fluctuating magnetic fields generated by FGT (see Supplementary Information Note 4 for details). Due to the strong perpendicular magnetic anisotropy, the minimum magnon energy of FGT is larger than the ESR frequencies of ${V}_{{{{{{\rm{B}}}}}}}^{-}$ spin defects in our experimentally accessible magnetic field range, hence, the measured spin relaxation is driven by the longitudinal spin fluctuations of FGT, which is further related to the static longitudinal magnetic susceptibility *χ*_0_ and the diffusive spin transport constant *D*^46,60,61^. When temperature is away from the quantum critical point, magnetic fluctuations in FGT are largely suppressed due to its vanishingly small magnetic susceptibility, leading to negligible spin relaxation rate ${\Gamma }_{{{{{{\rm{M}}}}}}}$ of ${V}_{{{{{{\rm{B}}}}}}}^{-}$ defects (Fig. 5a, b). In contrast, we observed significantly enhanced spin relaxation rate during the magnetic phase transition of FGT (Fig. 5c–e), which we attribute to the increase of the magnetic susceptibility of FGT around the Curie temperature^62^. When *T* is above 200 K, spin fluctuations remain active in FGT due to the finite spin-spin correlation in the paramagnetic state^63^. The observed spatially varying magnetic fluctuations over the exfoliated FGT flake could be induced by inhomogeneities in magnetic susceptibility, spin diffusion constant, and exchange coupling strength. Figure 5h summarizes the temperature dependence of the spatially averaged spin relaxation rate ${\Gamma }_{{{{{{\rm{M}}}}}}}$ with a peak value of 36 kHz around the Curie temperature, consistent with the ferromagnetic phase transition of FGT^62^. Invoking a theoretical model developed in Ref. 60, the longitudinal magnetic susceptibility *χ*_0_ and spin diffusion constant *D* of the exfoliated FGT flake is extracted to be (1.5 ± 0.2) × 10^−2^ emu cm^−3^ Oe^−1^ and (1.7 ± 0.3) × 10^−5^ m^2^/s at 189 K (see Supplementary Information Note 4 for details).

## Discussion

In summary, we have demonstrated ${V}_{{{{{{\rm{B}}}}}}}^{-}$ spin defects in hBN as a local probe to image magnetic phase transitions and spin fluctuations in the archetypical van der Waals ferromagnet FGT at the nanoscale. The spatially resolved wide-field magnetometry results reveal the characteristic evolution behavior of magnetic domains during the phase transition of FGT. By using ${V}_{{{{{{\rm{B}}}}}}}^{-}$ spin relaxometry techniques, we are also able to access the spin fluctuations in the FGT flake, whose magnitude reaches a maximum value around the Curie temperature. Our results illustrate the appreciable capability of ${V}_{{{{{{\rm{B}}}}}}}^{-}$ spin defects hosted by hBN of investigating local magnetic properties of layered materials in van der Waals heterostructure formats. The presented measurement platform also shows optimal field sensitivity with nanoscale sensor-to-sample distance^15,16,64^, offering new opportunities for advancing the state of the art of existing quantum sensing technologies. While the current study is conducted using wide-field magnetometry with a spatial resolution set by the optical diffraction limit, we anticipate that the spatial sensitivity of ${V}_{{{{{{\rm{B}}}}}}}^{-}$ spin defects could potentially reach the tens of nanometers regime by utilizing single-spin defects and developing scanning microscopy measurement schemes^2,44,50,57,65,66^, opening the possibility of uncovering detailed microscopic features in a broad range of 2D material systems.

## Methods

### Materials and device fabrication

The hBN and FGT crystals used in this study were commercially available from 2D Semiconductors. Thin samples were mechanically exfoliated onto Si/SiO_2_ (285 nm) substrates. hBN flakes were subsequently irradiated by Helium ions with an energy of 5 keV and a dose of 5 $\times$ 10^13^ cm^−2^. Irradiated hBN flakes with desirable lateral shape and dimensions are selected for fabricating FGT/hBN bilayer devices. FGT/hBN stack was prepared using the standard polydimethylsiloxane stamp process^67^. Selected flakes were picked up one-by-one by a stamp consisting of a thin layer of polycarbonate on polydimethylsiloxane, then released onto an Au transmission line pre-patterned on a separate Si/SiO_2_ (285 nm) substrate. The residual polycarbonate on the device was dissolved in chloroform before measurements. All the device fabrication processes involving handling FGT flakes were performed inside a glovebox filled with argon to minimize environmental degradation. Multiple control samples have been prepared and tested to ensure the reproducibility of the presented results (see Supplementary Information Note 5 for details).

### Pulsed ODMR and spin relaxometry measurements

Pulsed ODMR and spin relaxometry measurements were performed by a wide-field microscope. The prepared FGT/hBN heterostructure was positioned in a closed-cycle optical cryostat allowing for measurements from 4.5 K to 350 K. Microsecond-long green laser pulses used for spin initialization and readout were generated by an electrically driven 515-nm laser. The laser beam spot width after passing the objective was about 25 ${{{{{\rm{\mu }}}}}}$m $\times$ 25 ${{{{{\rm{\mu }}}}}}$m, and was subsequently focused on the hBN layer. Fluorescence of ${V}_{{{{{{\rm{B}}}}}}}^{-}$ spin defects was imaged using a CMOS camera. Pulses to drive the green laser and to trigger the camera exposure were generated by a programmable pulse generator. Continuous microwave currents were generated using Rohde & Schwarz SGS100a and/or Rohde & Schwarz SMB100a signal generators. 100-nanosecond-long microwave current pulses were generated by sending the continuous microwave currents to a microwave switch (Minicircuits ZASWA-2-50DR+) electrically controlled by a programmable pulse generator. The microwave pulses were sent through a microwave combiner (Mini-Circuits ZB3PD-63-S+) and amplified by $+$50 dB (Mini-Circuits ZHL-25W-63+) before being delivered to the on-chip Au stripline. The external magnetic field was generated by a cylindrical NdFeB permanent magnet attached to a scanning stage. The field sensitivity and other merits of the presented ODMR measurement platform are discussed in Supplementary Information Note 6 in detail.

## Supplementary information

Supplementary Information

## Data Availability

All data supporting the findings of this study are available from the corresponding author on reasonable request.

## Code availability

All code not included in the paper are available upon reasonable request from the corresponding authors.
