# Supplementary material for: Wide field imaging of van der Waals ferromagnet Fe3GeTe2 by spin defects in hexagonal boron nitride
Source: Nat Commun. 2022 Sep 13;13:5369. doi: 10.1038/s41467-022-33016-2 (PMC9470674; doi:10.1038/s41467-022-33016-2)
Supplement: Supplementary file 1 — Supplementary Information [file 41467_2022_33016_MOESM1_ESM.pdf]

Supplementary Information for Wide Field Imaging of van der Waals Ferromagnet Fe_3GeTe_2 by Spin Defects in Hexagonal Boron Nitride

Mengqi Huang,[1] Jingcheng Zhou,[1] Di Chen,[2,3] Hanyi Lu,[1] Nathan J. McLaughlin,[1] Senlei Li,[1] Mohammed Alghamdi,[4] Dziga Djugba,[1] Jing Shi,[4] Hailong Wang,[5] Chunhui Rita Du[1,5]

[1]Department of Physics, University of California, San Diego, La Jolla, California 92093
[2]Department of Physics, University of Houston, Houston, Texas 77204
[3]Texas Center for Superconductivity, University of Houston, Houston, Texas 77204
[4]Department of Physics and Astronomy, University of California, Riverside, California 92521
[5]Center for Memory and Recording Research, University of California, San Diego, La Jolla, California 92093

Supplementary Note 1. Thickness characterization of Fe_3GeTe_2/ hexagonal-boron-nitride device

Supplementary Fig. 1a shows an optical microscope image of a prepared Fe_3GeTe_2(FGT)/hexagonal boron nitride(hBN) heterostructure for wide-field imaging measurements. We used Atomic Force Microscopy (AFM) to characterize the thickness of individual layers. The dotted arrows in Supplementary Fig. 1a show the location and direction of AFM scans to characterize the thickness of hBN and FGT flakes. The scanning direction of the AFM tip was set to be near-perpendicular to all steps to accurately measure the relative thickness. AFM results are presented in Supplementary Figs. 1b and 1c, from which the thickness of the exfoliated hBN and FGT flakes were measured to be 98 and 86 nm, respectively.

Supplementary Note 2. Magneto-transport characterizations of exfoliated Fe_3GeTe_2 flakes

To characterize the magneto-transport properties of Fe_3GeTe_2(FGT), we mechanically transferred exfoliated FGT flakes onto separate Si/SiO_2 substrates with prefabricated Au electrodes for anomalous Hall measurements. Supplementary Fig. 2a shows an optical microscope image of a prepared device. The thickness of the FGT flake was characterized to be 114 nm, similar to that of the sample used in wide-field imaging measurements. An hBN flake was used to encapsulate the FGT flake to minimize environmental effects. Hall resistance R_H of the exfoliated FGT flake is characterized by a standard four-point measurement scheme with an external magnetic field B_{ext} applied along the out-of-plane direction. Supplementary Fig. 2b shows a series of hysteresis loops of R_H measured at different temperatures. Below 100 K, one can see that the R_H loops are largely square with the coercive field H_c monotonically increasing with decreasing temperature. When T = 10 K, H_c is above 3000 Oe, demonstrating strong perpendicular magnetic anisotropy of FGT. From 120 K to 190 K, the measured R_H loops become tilted and deviate from the square shape while H_c is dramatically reduced due to the enhanced thermal fluctuations. The anomalous Hall feature of the R_H loops fully disappears above 200 K. Supplementary Fig. 2c plots temperature dependence of the magnitude of the R_H loops, which is characterized by the difference between the two saturated values in the positive and negative field regimes. The Curie temperature is estimated to be ~200 K, in qualitative agreement with previous studies and our wide-field imaging results.[1–3]

Supplementary Note 3. Reconstruction of magnetization maps of an Fe_3GeTe_2 flake

A V_B^- spin defect in hBN is formed by a negatively charged boron vacancy surrounded by three nitrogen atoms, as shown in Fig. 1c in the manuscript. The negatively charged V_B^- spin defect has an $S = 1$ electron spin and serves as a spin-triplet quantum system. A magnetic field B_{tot} along the spin polarization direction will induce the Zeeman splitting of the spin states of V_B^- spin defect, and the electron spin resonance (ESR) frequency $f_\pm$ for $m_s = \pm1$ states can be expressed as:[4,5]

$$f_\pm = D_0 \pm \sqrt{E_0^2 + \left(\tilde{\gamma} B_{tot} / 2\pi\right)^2}, \quad (1)$$

where the zero-field splitting D_0 is 3.47 GHz at 295 K, the off-axial zero field splitting parameter E_0 is 50 MHz, and the gyromagnetic ratio of the V_B^- spin center $\tilde{\gamma}/2\pi$ is 2.8 MHz/G.[4,5] Therefore, the local magnetic field B_{tot} experienced by V_B^- defects can be obtained by measuring the splitting of the ESR frequencies:

$$B_{\rm tot} = \frac{\pi(f_+ - f_-)}{\tilde{\gamma}}. \tag{2}$$

Here we neglect the off-axial term E_0, which is much smaller than the Zeeman splitting term. In our measurements, $B_{\rm tot}$ contains contributions from both external magnetic field $B_{\rm ext}$ along z axis and the z component of the static stray field $B_{\rm F}$ generated by the Fe_3GeTe_2 (FGT) flake:

$$B_{\rm tot} = B_{\rm ext} + B_{\rm F}. \tag{3}$$

By subtracting the contribution of the external magnetic field $B_{\rm ext}$, stray field $B_{\rm F}$ generated by the FGT flake can be obtained as shown in Fig. 2b in the manuscript. Based on the obtained stray field results, we can reconstruct the 2D magnetization maps of the FGT flake using the method described below.

The coordinate system used for numerical data analysis is shown in Supplementary Fig. 3, where the surfaces of the hBN and FGT flakes lie in the x-y plane, and the ensemble of $V_{\rm B}^-$ spin defects are located in the plane of $z = 0$. Distance from the $V_{\rm B}^-$ spin defects to the top surface of the FGT flake is d, and the FGT sample with of a thickness of $t_{\rm FGT}$ occupies the space Ω with $-d - t_{\rm FGT} \leq z \leq -d$. In the global frame, the stray field distribution $\mathbf{B}_{\rm F}(\mathbf{R})$ is related to the magnetization distribution $\mathbf{M}(\mathbf{R}')$ of the FGT flake according to the magnetic dipole-dipole interaction in the following way:[6–8]

$$\mathbf{B}_{\rm F}(\mathbf{R}) = \int_{\Omega} d^3\mathbf{R}' \mathcal{D}(\mathbf{R},\mathbf{R}')\mathbf{M}(\mathbf{R}'), \tag{4}$$

where $\mathcal{D}(\mathbf{R},\mathbf{R}') = -\nabla_{\mathbf{R}}\nabla_{\mathbf{R}'}\left(1/|\mathbf{R}-\mathbf{R}'|\right)$ is the magnetostatic Green's function tensor between coordinates $\mathbf{R} = (x, y, z)$ and $\mathbf{R}' = (x', y', z')$. Note that magnetic stray field is related to magnetization of the sample through an integral with the Green's function tensor. In order to get a proportional relationship, we take the 2D Fourier transform:

$$\begin{aligned} \mathbf{B}_{\rm F}(\mathbf{k}) &= \int \mathbf{B}_{\rm F}(\mathbf{R}) e^{i(k_x x + k_y y)} dxdy, \\ \mathbf{M}(\mathbf{k}, z') &= \int \mathbf{M}(\mathbf{R}') e^{i(k_x x' + k_y y')} dx'dy', \end{aligned} \tag{5}$$

where $\mathbf{k} = (k_x, k_y, 0)$. When the z-axis translational symmetries are present, $\mathbf{M}(\mathbf{k}, z') = \mathbf{M}(\mathbf{k})$. According to the convolution theorem, the magnetic stray field $\mathbf{B}_{\rm F}$ at positions of spin defects ($z = 0$) can be written as:

$$\mathbf{B}_{\rm F}(\mathbf{k}) = \int D(\mathbf{k}, z')\mathbf{M}(\mathbf{k})dz', \tag{6}$$

where $D(\mathbf{k}, z')$ is the Green's function tensor in the Fourier space. Due to the spontaneous out-of-plane anisotropy of FGT, the magnetization is perpendicular to the sample surface: $\mathbf{M}(\mathbf{k}) = M(\mathbf{k})\hat{\mathbf{z}}$. Thus, the z component of the magnetic stray field $B_{\rm F}(\mathbf{k})$ can be expressed as:

$$\begin{aligned} B_{\rm F}(\mathbf{k}) &= \int_{-(t_{\rm FGT}+d)}^{-d} D_{zz}(\mathbf{k}, z')M(\mathbf{k})dz' \\ &= 2\pi\left[e^{-dk} - e^{-(t_{\rm FGT}+d)k}\right]M(\mathbf{k}), \end{aligned} \tag{7}$$

where $D_{zz}(\mathbf{k},z')=2\pi k\exp(kz')$ is the element of the Green's function tensor in Fourier space which relates the z component of $\mathbf{M}(\mathbf{k})$ to the z component of $\mathbf{B}_F(\mathbf{k})$.[6–8] Because the magnetic stray field $B_F(\mathbf{k})$ along z-axis is proportional to the magnetization $M(\mathbf{k})$ in the Fourier space, we can extract the magnetization distribution in the Fourier space through the measured magnetic stray field B_F based on Eq. (7). Last, we introduce an inverse Fourier transform to reconstruct magnetization in the real space:

$$M(\mathbf{R}) = M(x,y) = \frac{1}{(2\pi)^2}\int M(\mathbf{k})e^{-i(k_x x+k_y y)}dk_x dk_y. \tag{8}$$

Using the method discussed above, Fig. 2c in the manuscript shows an example of the reconstructed magnetization map of FGT sample at 6 K. The obtained FGT magnetization decreases with increasing temperature and the Curie temperature T_c is obtained to be ~ 200 K, consistent with the previous work.[1–3]

Supplementary Note 4. Inferring spin diffusion constant and longitudinal magnetic susceptibility of a Fe_3GeTe_2 flake from spin relaxometry measurements

In this section, we provide the details to extract spin diffusion constant and static longitudinal magnetic susceptibility of an FGT sample from spin relaxometry measurements. Considering the fact that the magnon gap of FGT is larger than the ESR frequency of V_B^- spin defects, the measured spin relaxation $\Gamma_M(f)$ of V_B^- at the frequency f is driven by longitudinal spin fluctuations in FGT which can be described through the two-magnon noise model:[6,7,9]

$$\Gamma_M(f) = \frac{1}{2\pi f\beta}G(\theta)\int_{-(t_{FGT}+d)}^{-d}dz\int_0^\infty dk k^3 e^{2kz}\chi''(k,f), \tag{9}$$

where $\beta = 1/k_B T$, k_B is the Boltzmann constant, T is the temperature, and $\chi''(k,f)$ is the imaginary part of the dynamical longitudinal spin susceptibility. The geometric factor $G(\theta)$ can be expressed as:

$$G(\theta) = \frac{(\gamma\tilde{\gamma})^2\pi}{2}(5-\cos 2\theta), \tag{10}$$

where $\theta = 0$ is the angle between the spin polarization direction of V_B^- defect and the magnetic easy axis of the FGT sample flake, and $\tilde{\gamma}$ and γ are the gyromagnetic ratio of V_B^- defects and FGT flake, respectively. To extract the dynamical longitudinal spin susceptibility $\chi''(k,f)$, we describe this system by a diffusion equation. Assuming U(1) symmetry, the diffusion equation for spin oriented along the direction of the magnetic order parameter s^z can be written as:[7,10]

$$\partial_t s^z + \nabla\cdot\mathbf{j}_s = -\frac{1}{\tau_s}\left(s^z - \chi H\right). \tag{11}$$

Here, we have introduced the spin-relaxation time τ_s and the spin current $\mathbf{j}_s = -\sigma\nabla\mu$, where σ is the spin conductivity, $\mu = \chi^{-1}s^z - H$ is the spin chemical potential, χ is the static uniform longitudinal spin susceptibility, and H is an external perturbation thermodynamical conjugate to the spin density. By introducing diffusion coefficient $D = \sigma/\chi$, we have:

$$\partial_t s^z - D\nabla^2 s^z + D\chi\nabla^2 H = -\frac{1}{\tau_s}\left(s^z - \chi H\right). \tag{12}$$

By introducing the Fourier transform $s^z\left(\mathbf{k}, f\right) = \int e^{i(\mathbf{k}\cdot\mathbf{r} - 2\pi f t)} s^z\left(\mathbf{r}, t\right) d^2\mathbf{r}dt$, we have:

$$s^z\left(\mathbf{k}, f\right) = \frac{\chi\left(Dk^2 + 1/\tau_s\right)}{2\pi i f + \left(Dk^2 + 1/\tau_s\right)} H. \tag{13}$$

Thus, the imaginary part of the dynamical longitudinal spin susceptibility can be written as:

$$\chi''\left(k, f\right) = \frac{2\pi\chi\left(Dk^2 + 1/\tau_s\right) f}{\left(2\pi f\right)^2 + \left(Dk^2 + 1/\tau_s\right)^2}. \tag{14}$$

Combining Eq. (9) and Eq. (14), and considering the limit of long relaxation time ($\tau_s \to \infty$), the spin relaxation rate of V_B^- spin defects driven by longitudinal spin fluctuations in a proximate magnet can be expressed as:

$$\Gamma_M(f) = \frac{G(\theta)}{2\beta} \int dk k^2 e^{-2kd} \left(1 - e^{-2kt_{FGT}}\right) \frac{\chi D k^2}{\left(2\pi f\right)^2 + \left(Dk^2\right)^2}. \tag{15}$$

Note that we have introduced the magnetic longitudinal susceptibility $\chi_0 = \gamma^2\chi$ in order to express the susceptibility in a typical unit of emu·cm^{-3}·Oe^{-1}.

Supplementary Fig. 4a shows a 2D map of the relaxation rate of V_B^- spin defects measured at 189 K. The applied perpendicular magnetic field B_{ext} is 590 Oe, corresponding to an ESR frequency of 1.9 GHz. We note that the extracted spin relaxation rate Γ contains the intrinsic component Γ_0 and the extrinsic component Γ_M driven by the longitudinal spin fluctuations in the proximate FGT flake: $\Gamma = \Gamma_0 + \Gamma_M$. Γ_0 can be obtained by measuring the relaxation rate of V_B^- defects positioned away from the magnetic flake. By subtracting Γ_0 from Γ, the spin relaxation rate Γ_M of V_B^- defects driven by spin fluctuations in the proximate magnetic flake can be obtained, as shown in Supplementary Fig. 4b. By fitting the measured spatially averaged relaxation rates of Γ_M to Eq. (15), spin diffusion constant D of FGT is obtained to be (1.7 $\pm$ 0.3) ×10^{-5} m^2/s at 189 K (Supplementary Fig. 4c). This value is consistent with the theoretical estimation of $D = v^2\tau/3$ by taking a magnon velocity $v \sim (J_S a)/\hbar \sim$ 3.9 km/s (J_S is the exchange coupling strength, a is the lattice constant, and $\hbar$ is the reduced Planck constant)[1] and a momentum scattering time $\tau \sim$ 3 ps. The static magnetic longitudinal susceptibility χ_0 of the FGT sample is extracted to be (1.5 $\pm$ 0.2) ×10^{-2} emu·cm^{-3}·Oe^{-1}, also in agreement with previous results.[11]

Supplementary Note 5. Extended wide-field imaging results and control measurements

Supplementary Figs. 5a-5f show sets of optically detected magnetic resonance (ODMR) spectra measured at V_B^- spin defects located right above the FGT flake in the prepared hBN/FGT device shown in Supplementary Fig. 1a. The external perpendicular magnetic field is set to be 142 G in these measurements and the temperature is varied from 6 K to 225 K. Note that we used pulsed ODMR measurement protocol to minimize the potential laser and microwave induced

broadening effect.[12] The magnitude of the magnetic stray field B_F can be extracted from the separation between the upper and lower electron spin resonance (ESR) frequencies. Supplementary Figs. 6a-6f show magnetic stray field B_F maps generated by the prepared hBN/FGT device at individual temperatures. The magnitude of the measured magnetic stray field B_F gets clearly enhanced in the area of the FGT flake, and B_F vanishes to zero above the Curie temperature of FGT. The presented results are in a sharp contrast with the data measured on a bare hBN flake shown in Supplementary Fig. 7, where vanishingly small magnetic stray field B_F is measured in the entire temperature range. Further spin relaxometry results measured on the bare hBN flake are shown in Supplementary Figs. 8a-8g. The measured intrinsic spin relaxation rate Γ_0 increases monotonically with the temperature (Supplementary Fig. 8h), and the characteristic singularity behavior across the Curie temperature of FGT is absent. These control experiments demonstrate that the observed variations in magnetic stray field B_F and spin relaxation rate Γ_M measured on the hBN/FGT device cannot be driven by some intrinsic effects of V_B^- spin defects in hBN. To further demonstrate the reproducibility of the presented results, we performed wide-field magnetometry measurements to image the magnetic phase transition of another hBN/FGT device shown in Supplementary Fig. 9a. The thickness of the hBN flake is ~66 nm characterized by AFM (Supplementary Fig. 9b) and the implantation depth of V_B^- spin defects is estimated to be 52 nm by Stopping and Range of Ions in Matter (SRIM) simulations (Supplementary Fig. 9c). The sensor-to-sample distance is estimated to be 14 nm for this hBN/FGT device. Supplementary Figs. 9d-9k show the reconstructed magnetization maps of the FGT flake measured at various temperatures with an external perpendicular magnetic field B_{ext} = 581 G. The exhibited temperature dependent magnetic behavior of the FGT flake is in qualitative agreement with the results shown in the main text, and is also consistent with previous results.[1–3]

Supplementary Note 6. Field sensitivity of boron-vacancy-based wide-field magnetometry and comparison with existing quantum sensing techniques

In this section, we discuss the field sensitivity of the presented boron-vacancy-based wide-field magnetometry method. The field sensitivity limit of the reported ODMR measurements using boron vacancy V_B^- can be estimated by the following equation:[13]

$$\eta_B = \frac{1}{\tilde{\gamma} C T_2^* \sqrt{\alpha R}}, \tag{16}$$

where $\tilde{\gamma}$ and T_2^* are the gyromagnetic ratio and spin-dephasing time of boron vacancy spin defects, respectively, C is the spin contrast, $\alpha \approx 1$ is the readout duty cycle, and R is the photon counting rate. Substituting the following parameters in our measurements into Eq. 16: $R = 1.9 \times 10^5$ s^{-1}, C = 0.1, and T_2^* = 105 ns, the magnetic field sensitivity η_B is estimated to be ~8 $\mu T/\sqrt{Hz}$, in qualitative agreement with the value reported in the previous work.[14] Note that this value is lower than that of nitrogen vacancy (NV) centers mainly due to the shorter T_2^*. Meanwhile, we would like to note that the ultimate field sensitivity of boron vacancy is expected to reach ~ 20 $nT/\sqrt{Hz}$ by optimizing the density of spin defects, optical contrast, and photon collection efficiency as reported in Ref. 5.

Next, we show that the off-axis magnetic field emanated from the FGT sample, to the first order, does not affect the magnetic stray field B_F measured in our experiments. Using the measured

FGT magnetization at 6 K as shown in manuscript Fig. 2c, we can estimate that the maximum off-axis magnetic field B_x is 18.50 G at the sample edge, and the parallel component (along the z-axis) of the magnetic stray field B_z is 40.24 G. Here we assume that the off-axis field from the FGT flake is mainly along the x-axis because the sample is in a rectangular shape with its short axis along the x-axis direction. Substituting these numbers together with the external magnetic field B_{ext} = 142.01 G, zero-field splitting (ZFS) parameter D_0 = 3.64 GHz, and off-axis ZFS parameter E_0 = 50 MHz into the effective spin Hamiltonian H of a boron vacancy spin defect:[15]

$$H = D_0 S_z^{\ 2} + E_0 (S_x^{\ 2} - S_y^{\ 2}) + \tilde{\gamma}(B_{ext} + B_z) S_z + \tilde{\gamma} B_x S_x, \tag{17}$$

where S_x, S_y, and S_z are spin-1 Pauli matrices, and $\tilde{\gamma}$ is the gyromagnetic ratio of a boron vacancy, the upper and lower ESR frequencies $f_{\pm}$ are obtained to be 4.15631 GHz and 3.13629 GHz by solving the eigenvalues of Eq. 17. If we do not consider the off-axis components in Eq. 17, the out-of-plane magnetic stray field generated by the FGT sample $B_F = \pi\,(f_+ - f_-)/\tilde{\gamma} - B_{ext}$ is calculated to be 40.14 G. Note that the difference between the calculated B_F and our experimentally measured B_z (40.24 G) is within the experimental sensitivity. As the temperature increases, the off-axis magnetic stray field will be even smaller due to reduced FGT magnetization, leading to a vanishingly small effect on our results over the entire measurement temperature range.

Lastly, we briefly discuss the advantages and potential of hBN in quantum sensing research study. First of all, hBN offers an attractive platform to establish nanoscale proximity between spin defects and layered 2D materials in van der Waals heterostructures. For the prepared hBN/FGT heterostructure device shown in Supplementary Fig. 9a, the vertical distance between the V_B^- spin defects and the top surface of FGT flake is estimated to be 14 nm, showing a clear advantage to that of the state-of-the-art scanning nitrogen-vacancy (NV) techniques with a typical NV-to-sample distance ranging from 50 nm to 100 nm.[16–19] We highlight that the sensor-to-sample distance could ultimately reach the atomic length scale by thinning hBN flakes down to the monolayer thickness regime. Secondly, in comparison with the conventional 3D counterparts, spin defects hosted by hBN exhibit improved versatility for implementing ultrasensitive quantum sensing of proximate objects and remarkable compatibility to device integration. For example, there are several technical challenges when applying the existing NV-based wide-field magnetometry techniques to study the local electrical and magnetic properties of 2D materials. If we transfer patterned diamond membranes or microchips containing NV centers on top of 2D materials studied, the NV-to-sample distance typically stays in the range from hundreds of nanometers to a few micrometers depending on the flatness and roughness of patterned diamond samples.[13,20] Such increased sensor-to-sample distance will reduce the sensitivity of spin defects to the local magnetic fields arising from the sample. In addition, the sensor-to-sample distance cannot be precisely controlled in this measurement configuration, which will inevitably complicate the data analysis. One can also transfer 2D materials on top of a diamond sample containing NV centers. However, many metallic or semiconducting 2D materials, such as Fe_3GeTe_2, $MnBi_2Te_4$, and $FeTe_xSe_{1-x}$, are not optically transparent, thus, the probing laser is required to be incident on the back surface of a micrometer-thick diamond membrane in order to access sufficiently strong NV photoluminescence signals. A clear drawback of this approach is the difficulty of diamond fabrication which involves complicated acid cleaning, diamond etching, and diamond cleaving processes. Therefore, the wide-field magnetometry system using spin defects in hBN shows certain advantages in these aspects.

Despite of the potential benefits discussed above, we are aware that hBN based quantum sensing technology remains at its infancy. Tremendous research efforts are underway to address the technical challenges involving 2D device fabrications and development of new sensing method/techniques. Meanwhile, we fully acknowledge the existing NV quantum sensing techniques. Apparently, NV centers also exhibit advantages on some aspects, such as excellent quantum coherence and established adaptability to scanning measurement scheme. We anticipate that the emerging spin defects in hBN will complement with NV centers to push the performance of quantum sensing techniques to the next level.

References

1. Deng, Y. et al. Gate-tunable room-temperature ferromagnetism in two-dimensional Fe_3GeTe_2. *Nature* **563**, 94–99 (2018).
2. Fei, Z. et al. Two-dimensional itinerant ferromagnetism in atomically thin Fe_3GeTe_2. *Nature Mater* **17**, 778–782 (2018).
3. Xu, J., Phelan, W. A. & Chien, C.-L. Large anomalous Nernst effect in a van der Waals ferromagnet Fe_3GeTe_2. *Nano Lett.* **19**, 8250–8254 (2019).
4. Gottscholl, A. et al. Initialization and read-out of intrinsic spin defects in a van der Waals crystal at room temperature. *Nat. Mater.* **19**, 540–545 (2020).
5. Gottscholl, A. et al. Spin defects in hBN as promising temperature, pressure and magnetic field quantum sensors. *Nat Commun* **12**, 4480 (2021).
6. van der Sar, T., Casola, F., Walsworth, R. & Yacoby, A. Nanometre-scale probing of spin waves using single electron spins. *Nat Commun* **6**, 7886 (2015).
7. Flebus, B. & Tserkovnyak, Y. Quantum-impurity relaxometry of magnetization dynamics. *Phys. Rev. Lett.* **121**, 187204 (2018).
8. Dovzhenko, Y. et al. Magnetostatic twists in room-temperature skyrmions explored by nitrogen-vacancy center spin texture reconstruction. *Nat Commun* **9**, 2712 (2018).
9. Wang, H. et al. Noninvasive measurements of spin transport properties of an antiferromagnetic insulator. *Sci. Adv.* **8**, eabg8562 (2022).
10. Fang, H., Zhang, S. & Tserkovnyak, Y. A generalized model of magnon kinetics and subgap magnetic noise. *Phys. Rev. B* **105**, 184406 (2022).
11. Wang, H. et al. Characteristics and temperature-field-thickness evolutions of magnetic domain structures in van der Waals magnet Fe_3GeTe_2 nanolayers. *Appl. Phys. Lett.* **116**, 192403 (2020).
12. Dréau, A. et al. Avoiding power broadening in optically detected magnetic resonance of single NV defects for enhanced dc magnetic field sensitivity. *Phys. Rev. B* **84**, 195204 (2011).
13. Scholten, S. C. et al. Widefield quantum microscopy with nitrogen-vacancy centers in diamond: Strengths, limitations, and prospects. *J. Appl. Phys.* **130**, 150902 (2021).
14. Gao, X. et al. High-contrast plasmonic-enhanced shallow spin defects in hexagonal boron nitride for quantum sensing. *Nano Lett.* **21**, 7708–7714 (2021).
15. Gottscholl, A. et al. Room temperature coherent control of spin defects in hexagonal boron nitride. *Sci. Adv.* **7**, eabf3630 (2021).
16. Sun, Q.-C. et al. Magnetic domains and domain wall pinning in atomically thin $CrBr_3$ revealed by nanoscale imaging. *Nat Commun* **12**, 1989 (2021).
17. Haykal, A. et al. Antiferromagnetic textures in $BiFeO_3$ controlled by strain and electric field. *Nat Commun* **11**, 1704 (2020).
18. Thiel, L. et al. Probing magnetism in 2D materials at the nanoscale with single-spin microscopy. *Science* **364**, 973–976 (2019).
19. Vélez, S. et al. High-speed domain wall racetracks in a magnetic insulator. *Nat Commun* **10**, 4750 (2019).
20. Schlussel, Y. et al. Wide-field imaging of superconductor vortices with electron spins in diamond. *Phys. Rev. Applied* **10**, 034032 (2018).

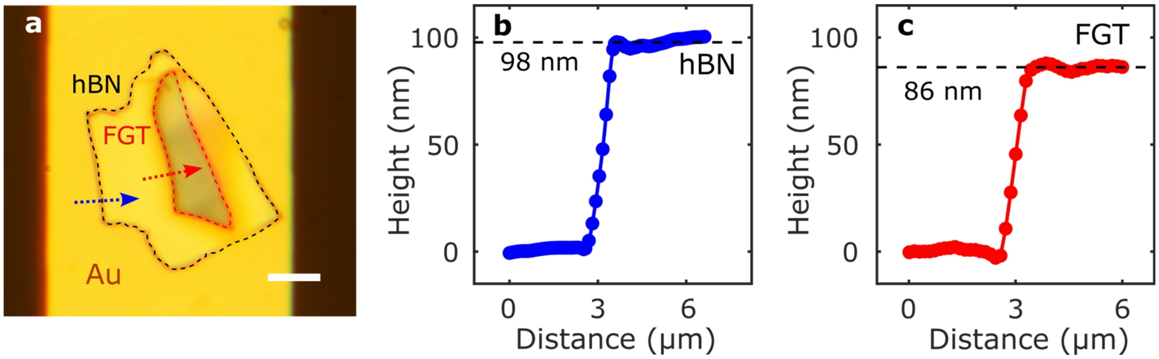

Supplementary Fig. 1. Thickness characterization of a prepared FGT/hBN heterostructure device. a Optical microscope image of the prepared device, where the FGT and hBN flakes are outlined by red and black dashed lines, respectively. Dotted arrows show the lines scanned by AFM tip to characterize the flake thickness, and the scale bar is 5 μm. **b, c** One-dimensional AFM scans from which the thicknesses of hBN and FGT flakes are measured to be 98 and 86 nm, respectively.

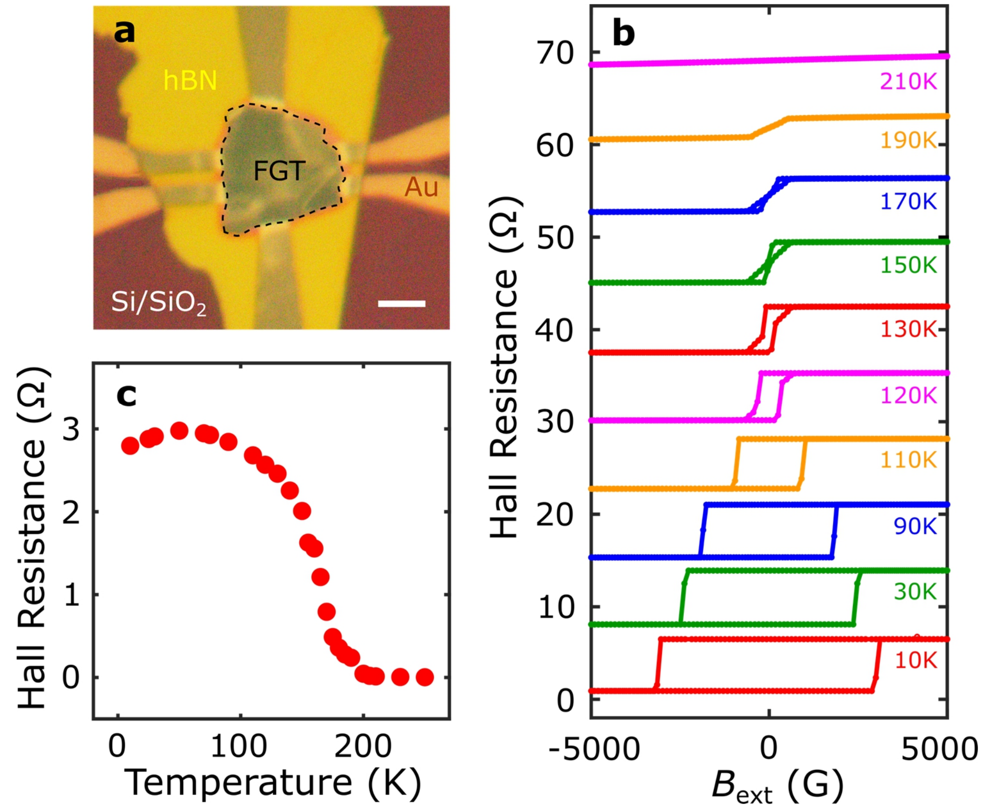

Supplementary Fig. 2. Magneto-transport characterizations of exfoliated FGT flakes. a Optical microscope image of a prepared FGT Hall device for transport measurements. Scale bar is 5 μm. **b** Hysteresis loops of Hall resistance of the FGT device measured at different temperatures. **c** Temperature dependence of anomalous Hall resistance of the FGT flake, from which the Curie temperature is estimated to be ~200 K.

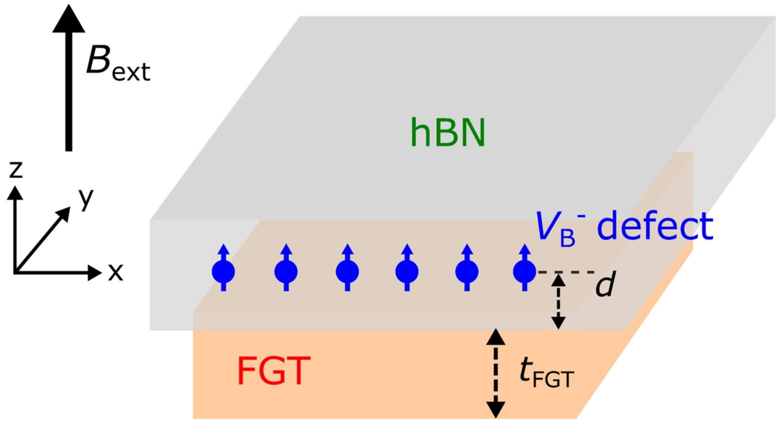

Supplementary Fig. 3. Reconstruction of two-dimensional magnetization maps from stray field results. Schematic and coordinate system used for numerical analysis. The surfaces of hBN and FGT flakes lie in the *x*-*y* plane, and the ensemble of V_B^- spin defects are located in the plane of $z = 0$. Distance between the V_B^- spin defects and the top surface of the FGT flake is *d*, and the thickness of FGT sample is t_{FGT}.

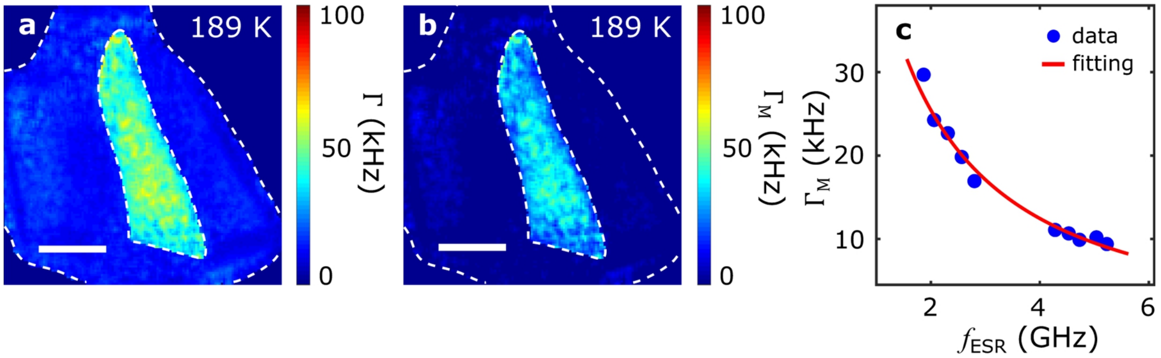

Supplementary Fig. 4. Extraction of longitudinal magnetic susceptibility and spin diffusion constant of an FGT flake. a 2D spin relaxation rate Γ map of V_{B}^{-} spin defects measured at 189 K. The ESR frequency of V_{B}^{-} spin defects f_{ESR} is set to be approximately 1.9 GHz with an external magnetic field B_{ext} = 590 G. The intrinsic relaxation rate Γ_0 can be obtained from the results of V_{B}^{-} defects positioned away from the magnetic flake. **b** 2D map of the extrinsic spin relaxation rate Γ_{M} driven by spin fluctuations in a proximate FGT sample. In Figs. **a** and **b**, white dashed lines outline the boundary of the FGT and hBN flakes, and the scale bar is 5 μm. **c** Frequency dependence of the spatially averaged extrinsic spin relaxation rate Γ_{M} of V_{B}^{-} spin defects positioned directly above the FGT flake. The experimental results (blue dots) are fit with the theoretical prediction (red curve), and the measurement temperature is 189 K.

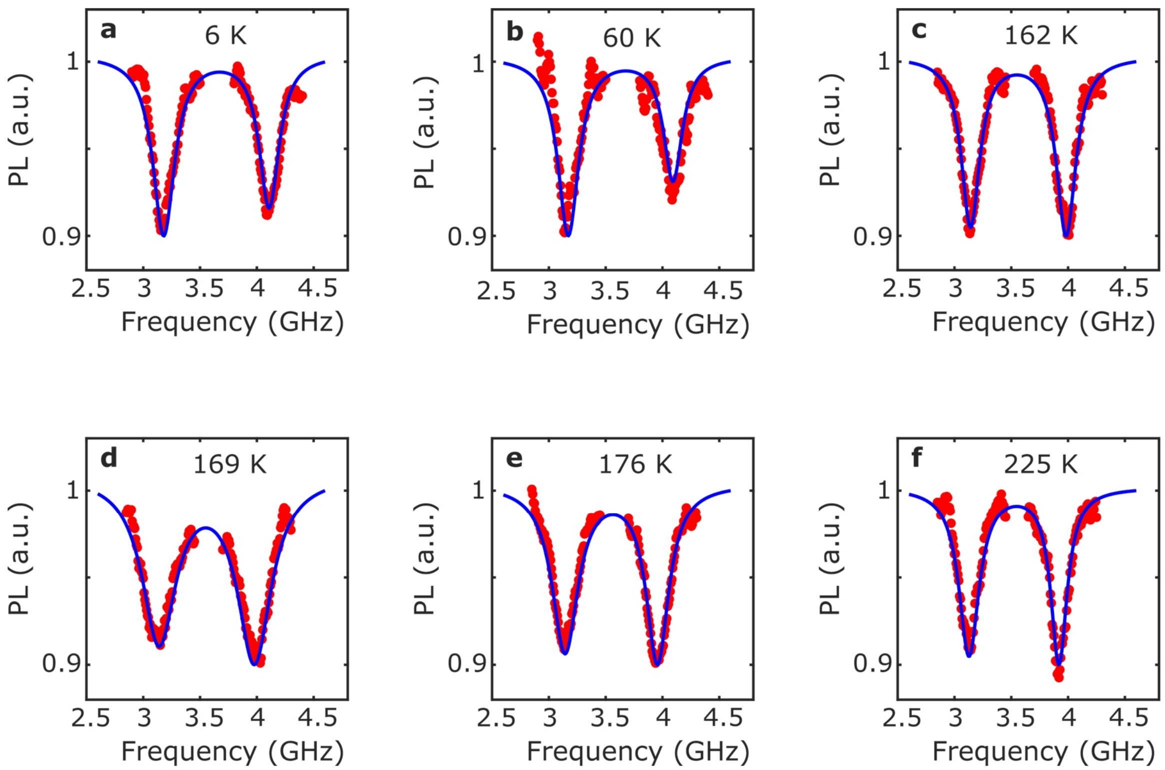

Supplementary Fig. 5. ODMR spectra measured at different temperatures. ODMR spectra measured on V_B^- spin defects located right above the FGT flake at temperatures of 6 K (**a**), 60 K (**b**), 162 K (**c**), 169 K (**d**), 176 K (**e**), and 225 K (**f**), respectively. The blue line represents a fitting of the data (red points). The external perpendicular magnetic field is 142 G for these measurements.

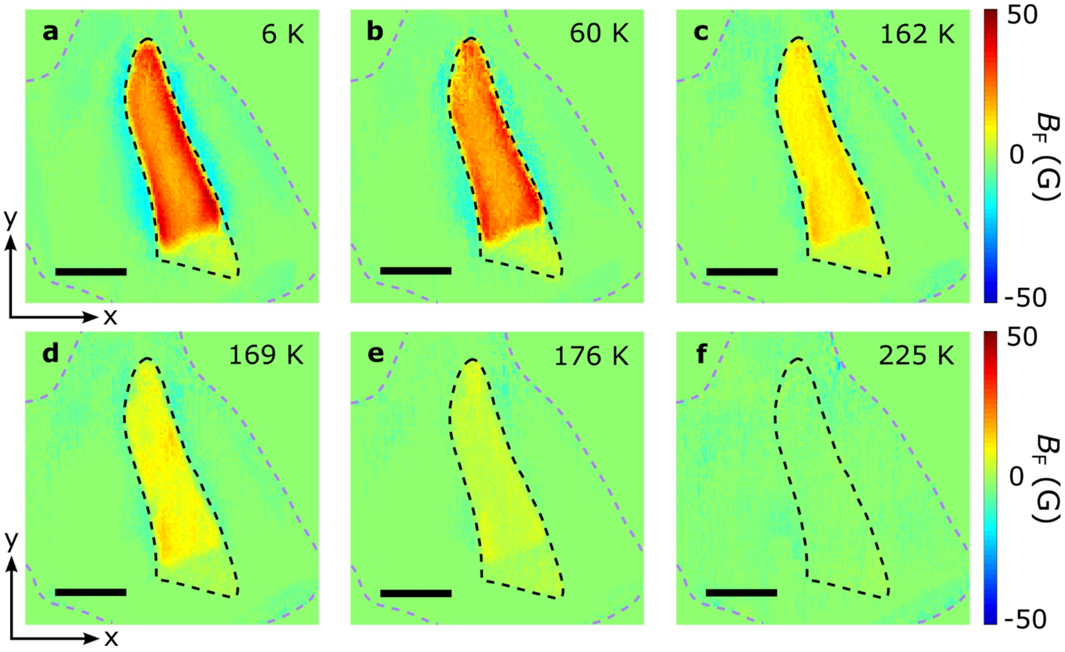

Supplementary Fig. 6. 2D imaging of magnetic stray field generated by an FGT flake. 2D maps of magnetic static stray field B_F of an exfoliated FGT flake measured with an external field B_{ext} = 142 G at temperatures of 6 K (**a**), 60 K (**b**), 162 K (**c**), 169 K (**d**), 176 K (**e**), and 225 K (**f**), respectively. The black and purple dashed lines outline the boundary of the FGT and hBN flakes, respectively, and the scale bar is 5 μm.

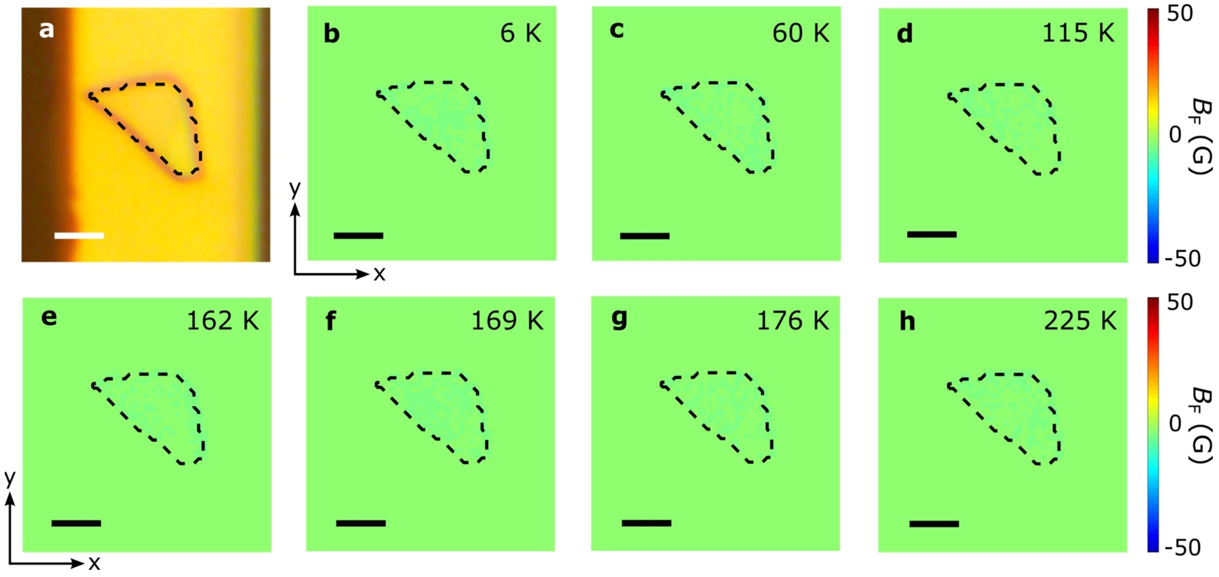

Supplementary Fig. 7. 2D imaging of magnetic stray field generated by a bare hBN flake. a Optical microscope image of a bare hBN flake containing boron vacancy V_B^- transferred on an Au stripline. The black dashed line outlines the boundary of the hBN flake. 2D maps of magnetic static stray field B_F generated by the hBN flake measured with an external field B_{ext} = 142 G at temperatures of 6 K (**b**), 60 K (**c**), 115 K (**d**), 162 K (**e**), 169 K (**f**), 176 K (**g**), and 225 K (**h**), respectively. The scale bar is 3 μm.

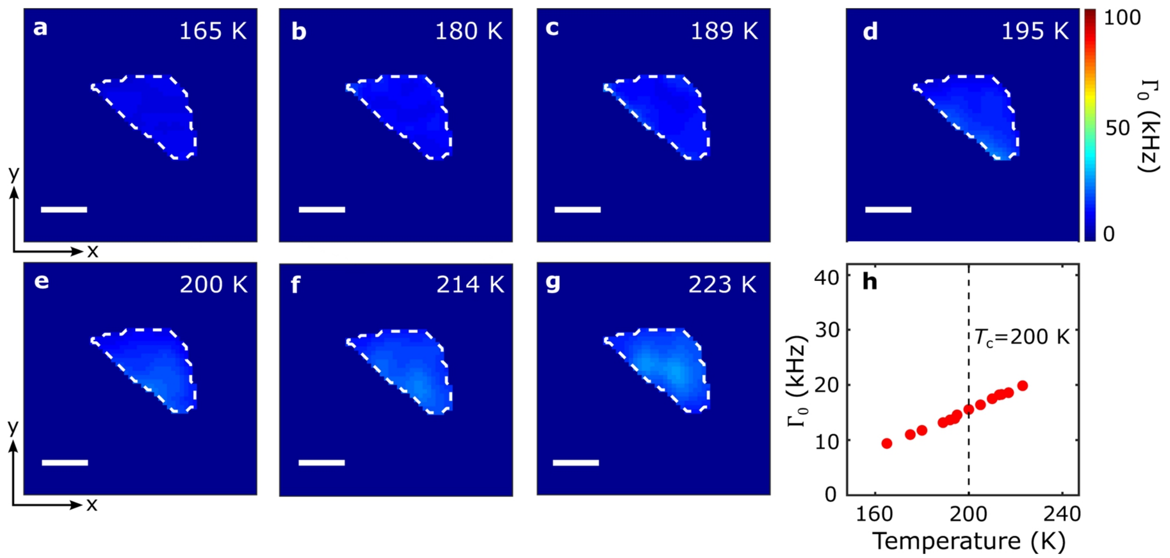

Supplementary Fig. 8. 2D mapping of intrinsic spin relaxation rate of boron vacancy in a bare hBN flake. 2D Maps of intrinsic spin relaxation rate of V_B^- spin defects measured at temperatures of 165 K (**a**), 180 K (**b**), 189 K (**c**), 195 K (**d**), 200 K (**e**), 214 K (**f**), and 223 K (**g**), respectively. The ESR frequency of V_B^- spin defects f_{ESR} is set to be approximately 1.9 GHz in these measurements with an external magnetic field B_{ext} = 590 G. The white dashed lines outline the boundary of the hBN flake, and the scale bar is 3 μm. **h** Temperature dependence of the spatially averaged intrinsic spin relaxation rate Γ_0 of V_B^- spin defects, showing a monotonic increase with temperature. The black dashed line marks the Curie temperature of the FGT flake.

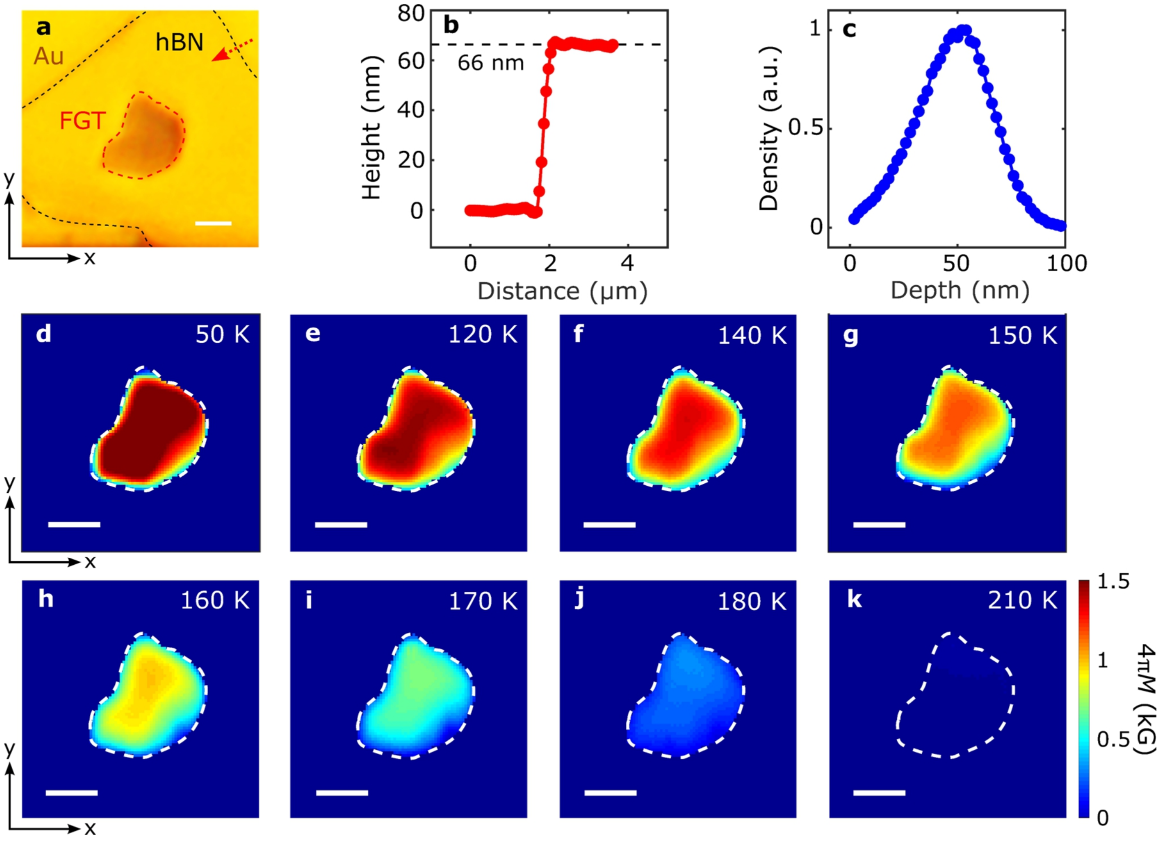

Supplementary Fig. 9. Quantum imaging of temperature dependence of FGT magnetization in a control sample. a Optical microscope image of a second FGT/hBN heterostructure device, where the FGT and hBN flakes are outlined by red and black dashed lines, respectively. The scale bar is 3 μm. **b** One-dimensional AFM scan from which the thicknesses of the hBN flake is characterized to be 66 nm. **c** Depth distribution of V_B^- spin defects created by Helium ion implantation with an energy of 5 keV. **d-k** Reconstructed magnetization ($4\pi M$) maps of the FGT flake at external magnetic field B_{ext} = 581 G and temperatures of 50 K (**d**), 120 K (**e**), 140 K (**f**), 150 K (**g**), 160 K (**h**), 170 K (**i**), 180 K (**j**), and 210 K (**k**), respectively. The white dashed lines outline the boundary of the exfoliated FGT flake, and the scale bar is 5 μm.
